# *In vivo* MRI with Concurrent Excitation and Acquisition using Automated Active Analog Cancellation

**DOI:** 10.1038/s41598-018-28894-w

**Published:** 2018-07-13

**Authors:** Ali Caglar Özen, Ergin Atalar, Jan G. Korvink, Michael Bock

**Affiliations:** 1 Department of Radiology, Medical Physics, Medical Center - University of Freiburg, Faculty of Medicine, University of Freiburg, Freiburg, Germany; 2 German Cancer Consortium Partner Site Freiburg, German Cancer Research Center (DKFZ), Heidelberg, Germany; 3 Department of Electrical and Electronics Engineering, Bilkent University, Ankara, Turkey; 4 Institute of Microstructure Technology, Karlsruhe Institute of Technology, Karlsruhe, Germany

## Abstract

Magnetic resonance imaging (MRI) provides excellent cross-sectional images of the soft tissues in patients. Unfortunately, MRI is intrinsically slow, it exposes patients to severe acoustic noise levels, and is limited in the visualization of certain tissues such as bone. These limitations are partly caused by the timing structure of the MRI exam which first generates the MR signal by a strong radio-frequency excitation and later acquires the weak MRI signal. Concurrent excitation and acquisition (CEA) can overcome these limitations, but is extremely challenging due to the huge intensity difference between transmit and receive signal (up to 100 dB). To suppress the strong transmit signals during signal reception, a fully automated analog cancellation unit was designed. On a 3 Tesla clinical MRI system we achieved an on-resonance analog isolation of 90 dB between the transmit and receive path, so that CEA images of the head and the extremities could be acquired with an acquisition efficiency of higher than 90% at sound pressure levels close to background noise. CEA with analog cancellation might provide new opportunities for MRI in tissues with very short T_2_ relaxation times, and it offers a silent and time-efficient MRI acquisition.

## Introduction

In conventional magnetic resonance imaging (MRI), the water proton MR signal is first excited by a strong radio-frequency (RF) pulse, and the generated transverse magnetization is later detected using a set of receiver coils. RF transmission and data acquisition are time-interleaved as the transmitted (Tx) radio frequency pulse would create an unwanted signal in the receive (Rx) coils that is many orders of magnitude larger than the weak MR signal. The time-interleaved Tx/Rx scheme effectively avoids the saturation of the amplifiers in the Rx system, but leads to undesired dead times in the pulse sequences which severely limit the detection of tissue with ultra-short T_2_.

Recently, pulse sequences with extremely short excitation and acquisition delays have been developed that partially overcome the signal loss from rapid T_2_ decay in tissues such as bone, cartilage, lung, teeth, or collagen fibers^1–6^ or in short-T_2_ nuclei such as ^23^Na^7,8^ or ^17^O^9,10^. These so-called ultra-short echo time (UTE) pulse sequences utilize very short (*T*_pulse_ < 100 µs) non-selective RF pulses followed by a 3D radial data acquisition (i.e., a pure frequency encoding scheme)^2^. Alternatively, sequences such as BLAST^11^, RUFIS^12^ – recently reappeared as ZTE^13^, and WASPI^14^, SPRITE^15^, PETRA^16^ and ramped-hybrid-encoding^17^ were proposed where the RF pulse is already applied when the gradients are switched on to further reduce TE and to increase the acquisition bandwidth. However, in all of these techniques an intrinsic delay is present between the end of the RF pulse and the beginning of the MR data acquisition, which is caused by the combination of coil ring down, Tx/Rx switching, and ADC filtering delays. Thus, tissues with T_2_ below the RF pulse length cannot be measured as the magnetization is already decayed when data acquisition starts.

Concurrent excitation and acquisition (CEA), on the other hand, truly acquires the MR signal during excitation, so that signals from tissue with ultra-short T_2_ can be sampled. Furthermore, with CEA a nearly 100% signal acquisition efficiency can be achieved because no time is lost on delays or signal encoding without data acquisition (as e.g. during phase encoding). Historically, CEA or continuous wave (CW) NMR methods were widely used, in which the MR signal was acquired during a frequency-modulated long RF pulse^18^: the discovery of NMR by Bloch^19^ was based on CW-NMR, and even the first MR image by Lauterbur^20^ was acquired with this technique. In 1974, it was shown that CW absorption spectra could also be acquired with a rapidly swept RF pulse (i.e., linear region of an adiabatic passage) that correlates with the spin response^21,22^, and the rapid scan correlation method was further improved for 3D MRI in solids^23^. In 2006, rapid scan correlation MRS was successfully adapted to MRI by dividing the frequency swept RF pulse into short segments with interleaved signal acquisitions to isolate Tx and Rx^24^. Although the SWIFT method provides a virtually simultaneous excitation and acquisition in MRI, the need for very fast Tx/Rx switches as well as short and accurate pulse segments made it challenging to realize on clinical MRI systems.

Recently, Idiyatullin and co-workers demonstrated that it is possible to implement signal acquisition during RF excitation using a hybrid coupler to decouple Tx and Rx coils^25^, and other CEA methods were also suggested^26,27^. A fundamental problem of CEA is Rx saturation through direct coupling of Tx energy into the Rx coils. Idiyatullin achieved an isolation between Tx and Rx that was sufficient to reduce the Tx-induced signals to the Rx dynamic range. A high field implementation of this setup was presented in^28^. Recently we proposed an active decoupling scheme with a second Tx coil which created a destructive RF signal at the Rx coil leading to a total isolation of up to 70 dB^29^. However, coupling stability during the measurement is crucial in all CEA methods. For example, digital cancellation methods using a theoretical Tx signal fail when coil loading changes due to patient motion. Thus, pick-up coils (PUCs) were introduced to monitor the Tx-induced signal^29,30^, but the active decoupling requires long manual adjustments of phases and amplitudes, and the decoupling performance is always dependent on the coil loading.

The isolation between Tx and Rx units is a well-known problem in full duplex telecommunication where signal transmission and reception occur simultaneously. In MRI, RF excitation and MR signal are located in the same frequency band, which is described as *in band full duplex* (IBFD). Methods for IBFD can be categorized into passive suppression, active suppression, and digital cancellation^31^. Passive suppression methods use antenna separation^32^, antenna decoupling^33^, and circulator isolation^34^, whereas active suppression methods employ actively controlled circuits to subtract a copy of the Tx signal from the Tx-induced leakage signal in the Rx coil^35^. Recently, a combined active analog and digital cancellation was proposed operating in real time^36^, the analog cancellation part of which was also implemented for CEA MRI with very high on-resonant decoupling^37^, and even *in vivo* CEA MRI could be demonstrated after manually-set analog cancellation^38^. However, the systems based on the manual adjustment of decoupling parameters and analog cancellation settings are extremely sensitive to motion of the subject, and changes in coil loading, thus are not suitable for *in vivo* measurements.

The CEA decoupling system designed in this work uses a unique real time feedback control between the analog cancellation circuit and the MR system to facilitate automated adjustment of the analog cancellation settings. The system combines three full duplex decoupling strategies: geometrical decoupling for passive suppression, an analog cancellation circuit for active cancellation, and digital cancellation. The feasibility of CEA MRI with this system was demonstrated at a clinical 3 T MRI system in both phantom and *in vivo* experiments.

## Methods

**Figure 1:**
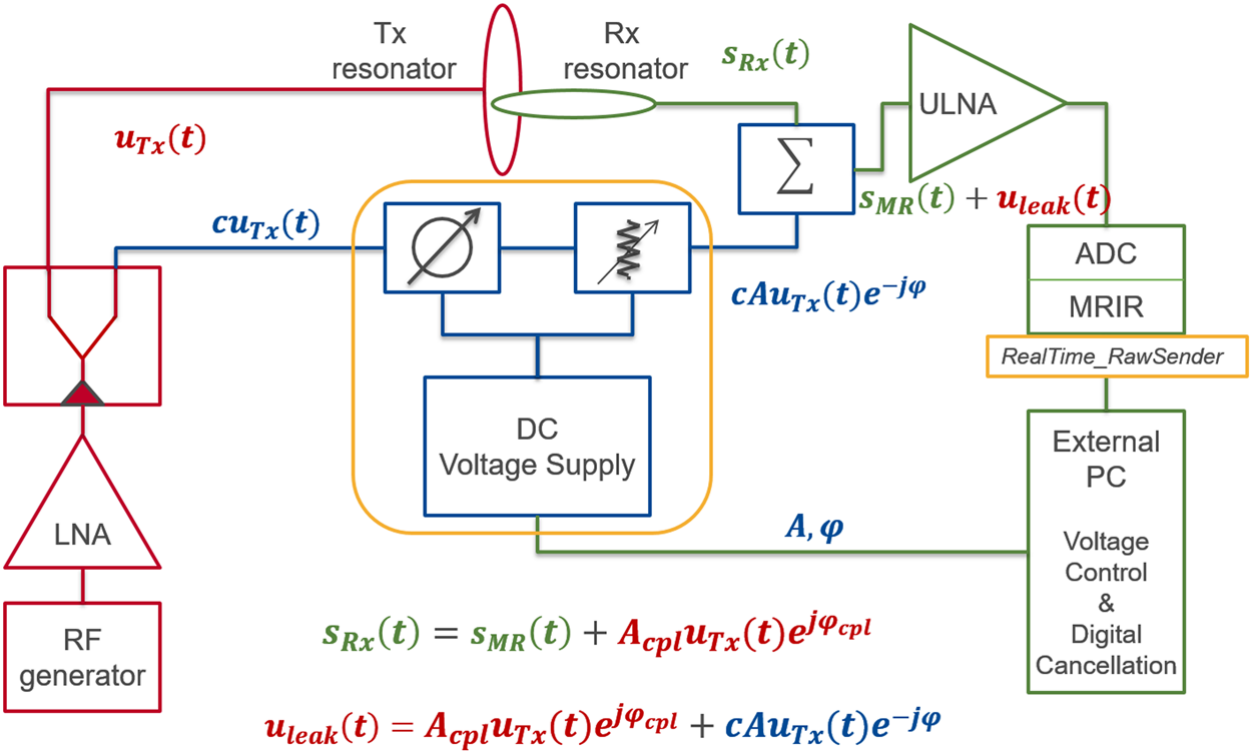
System setup for analog cancellation with real-time feedback. An external PC controls the phase and attenuation level of a small copy of the Tx signal to minimize the mean of the residual leakage signal. This control is based on serial communication via USB connection. Data transfer from the MR image reconstruction unit (MRIR) to the external PC is realized by TCP/IP protocol.

CEA with active analog cancellation and real time feedback was implemented in a clinical 3 T MRI system (Siemens AG, Erlangen, Germany). The setup of the total system is shown in Fig. 1: the Tx power is delivered to the main Tx coil via a directional coupler, where a small fraction of Tx signal is fed into a phase shifter and an attenuator. This modified copy of the Tx signal is then subtracted from the Rx signal to cancel the unwanted leakage of the Tx signal into the Rx coil. The settings of the phase shifter and the attenuator are continuously updated in a real-time feedback loop. To keep Tx noise low, a custom-made low noise amplifier (LNA) with 30 mW output power was used in the Tx chain. The Tx loop coil (ø = 15 cm) and the Rx loop coil (ø = 12 cm) were placed orthogonal to each other to achieve geometric decoupling (Fig. 1). In addition, a small pick-up loop coil (PUC) was placed in close proximity to the Tx coil to independently measure the Tx waveform.

### Analog Cancellation Circuit

The analog cancellation concept is based on subtracting a small copy of the Tx signal from the measured Rx signal at the receive coil output after proper phase and amplitude adjustment. A directional coupler (ZDC-10-1+, Mini-Circuits, Brooklyn, NY) divides the signal into two parts: ${u}_{{Tx}}(t)$ and ${{cu}}_{{Tx}}(t)$ where c = 0.28. During continuous RF excitation, a certain amount of Tx power is unavoidably coupled into the Rx coil, even if geometrical decoupling is used to minimize coil cross-talk. The task of the analog cancellation system is to match the phase ${\phi }_{{cpl}}$ and the amplitude ${A}_{{cpl}}$ of the Tx-induced signal at the Rx coil by searching for the optimal phase shift and attenuation (equation (1)). Then, the analog signals are subtracted using a power combiner (ADP-2-1 +, Mini-Circuits, Brooklyn, NY) before they are amplified and digitized in the subsequent Rx chain.

1 $$\begin{array}{rcl}{s}_{R}x(t) & = & {A}_{cpl}{u}_{Tx}{e}^{j{\phi }_{cpl}}+{s}_{MR}(t),\\ s(t) & = & {s}_{Rx}(t)-A{u}_{Tx}{e}^{j\phi }={s}_{MR}(t)+{{\rm{\min }}}_{A,\phi }{u}_{leak}.\end{array}$$

#### Voltage controlled phase shifter

**Figure 2:**
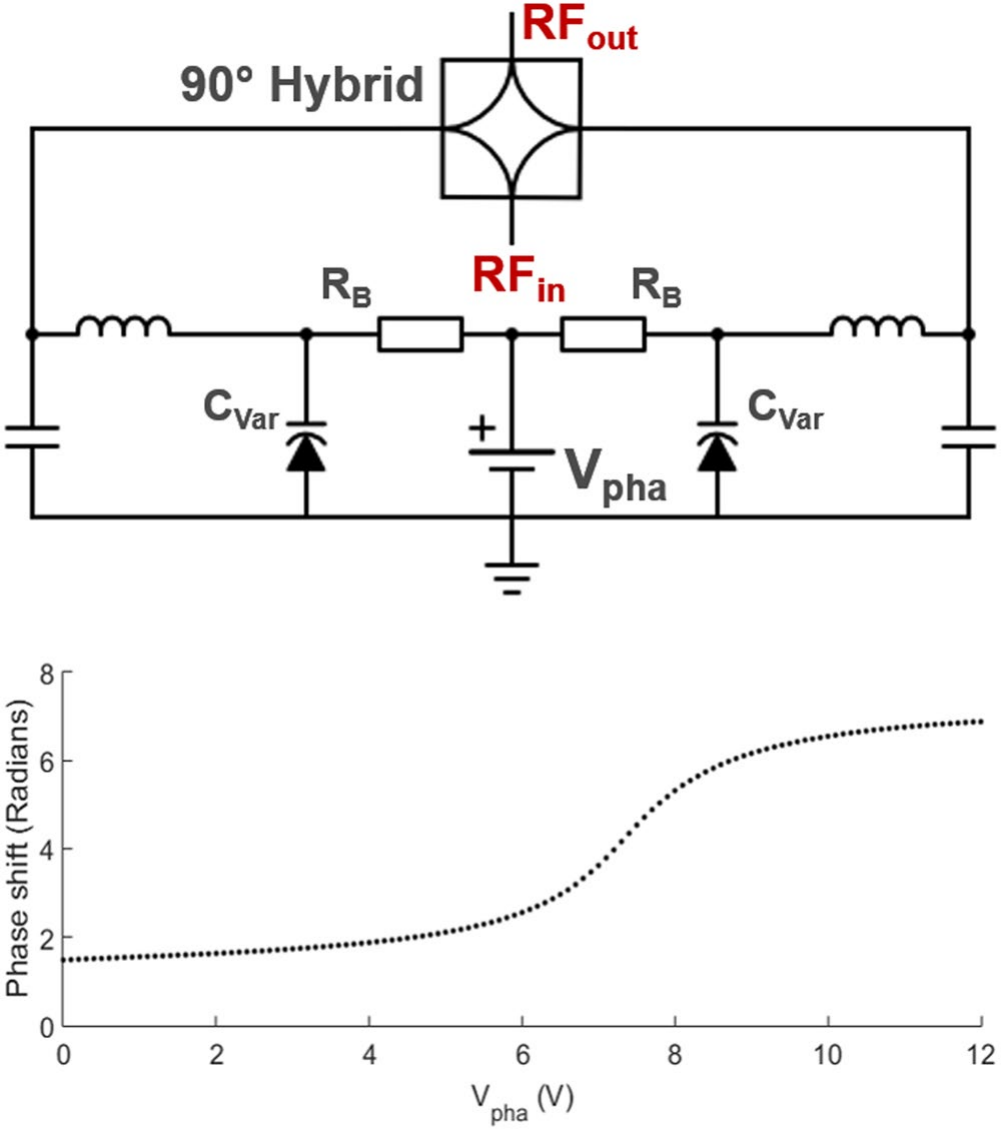
Voltage controlled phase shifter circuit schematic, and voltage versus phase plot. R_B_ stands for bias resistors. The phase shifter enables 280° coverage for 0 V < V_1_ < 12 V. Nonlinear behavior is taken into account by using variable voltage step sizes in automated feedback decoupling.

Analog phase shifting was realized by a reflection type phase shifter^39^, where impedances at the 2^nd^ and 3^rd^ ports of a 90° hybrid coupler (HE128MF, EMC Technology, Florida, USA) were changed using varactor diodes (BB640, Infenion Technologies, Munich, Germany). A digital programmable power supply (HMP4040, Rohde&Schwarz, Munich, Germany) was used to control voltage across, and thus the capacitance *C*_var_, of the diodes. According to the manufacturer data and own measurements, for a voltage sweep up to 25 V, the varactors’ capacitance monotonically increases to 75 pF. A simplified circuit schematic (i.e. DC feedthrough elements, DC blocking and bypass capacitors are excluded) and the resulting phase shift are shown in Fig. 2. The phase shifter unit has a transmission loss of −0.8 ± 0.35 dB within the given voltage range.

#### Voltage Controlled Attenuator

**Figure 3:**
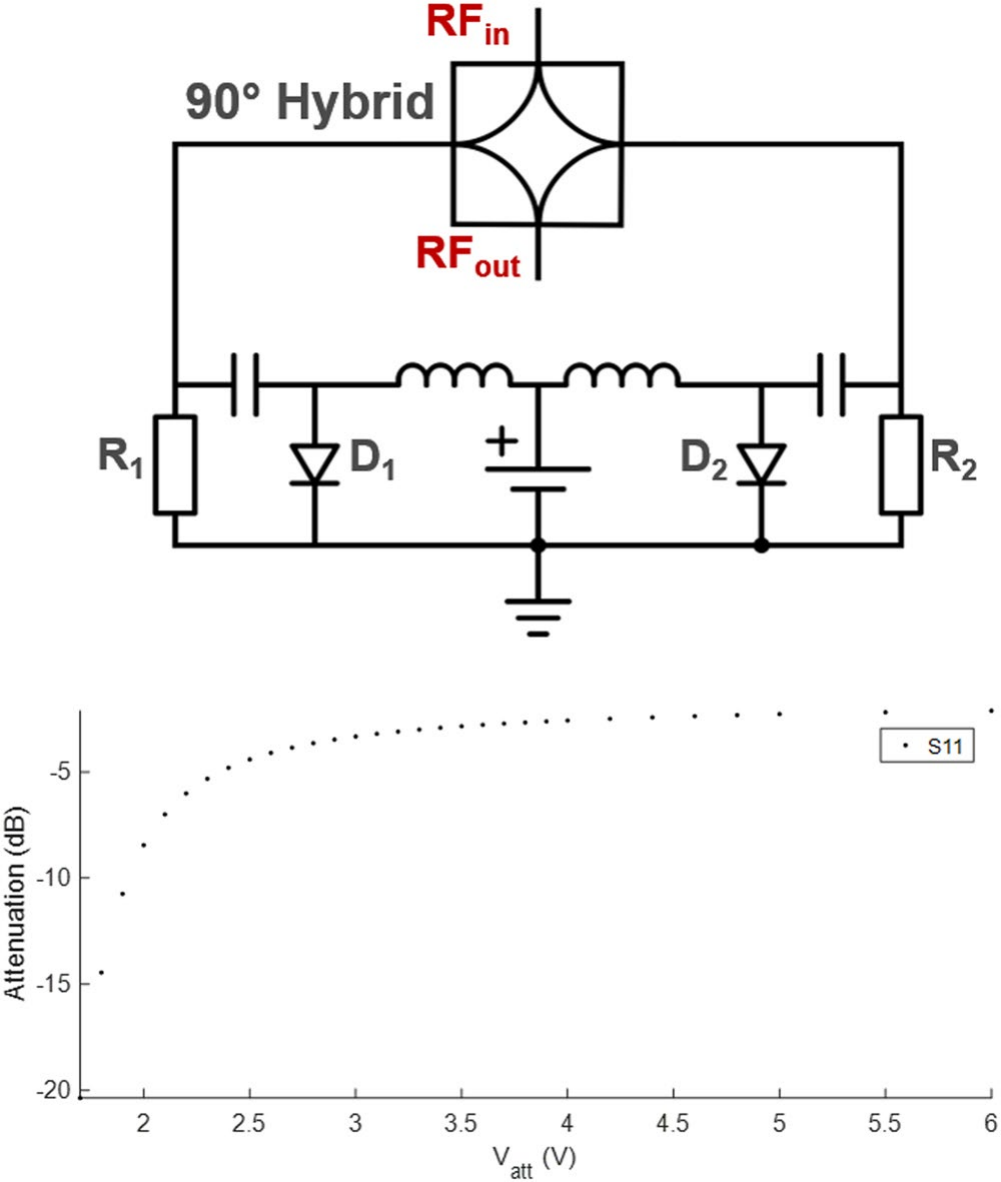
Voltage controlled attenuator circuit schematic, and voltage vs. attenuation on a logarithmic scale. R_1,2_ are the termination resistors of 50 Ω. The attenuation/voltage ratio is 1.2 ± 0.1 dB/V for V_att_ = 3.0–6.0 V. For V_att_ < 3.0 V, the slope of the attenuation vs. voltage curve is higher.

The variable attenuator is based on a 90° hybrid coupler network, the transmission ratio of which is controlled by symmetrically placed PIN diodes (MA4P7446F-1091, M/A-COM Technology Solutions, Inc., Lowell, USA). The PIN diodes are biased from a variable voltage source. PIN diodes behave as voltage controlled resistors. A simplified circuit schematic (i.e., DC feedthrough elements and bypass capacitors are excluded) is shown in Fig. 3. As *V*_*att*_ decreases, the impedance at the 2^nd^ and 3^rd^ ports approach 50 Ω, and thus the input signal is dissipated at the 2^nd^ and 3^rd^ ports. At *V*_*att*_ = 6.0 V, a minimum value of 1.3 dB attenuation was obtained (cf. Fig. 3). Between *V*_att_ = 3.0 V and 6.0 V the attenuation increases nearly linearly at about 1.2 ± 0.1 dB/V.

### Real time feedback operation

An external PC was used to implement real time feedback control of the phase shifter and attenuator units. Raw data was acquired with a special calibration pulse sequence that consists of a constant rectangular RF pulse of 4 ms duration, concurrent data acquisition, and a subsequent spoiler gradient. Raw data from each repetition was sent directly from the MRI receiver system to an external PC using real-time data transfer routines^40^ within the MR system image calculation environment (ICE). On the PC, a control program written in MATLAB was then changing the phase and amplitude settings of the analog cancellation circuit via a USB interface, which provided control voltages for the power supply of the analog cancellation circuit. The calibration sequence was repeated until the residual transmit leakage was below a user-defined threshold – typically, threshold values corresponding to 80 to 90 dB isolation were used.

During automated optimization, a gradient descent approach was used to change phase and amplitude values that minimize the objective function ${s}_{Rx}(A,\phi )=\sum _{n=1}^{N}{s}_{Rx}/N$, where N is the number sample points, and ${s}_{{Rx}}$ is the total received signal as described in Eq. 1. Initial voltage levels were determined experimentally to avoid receiver saturation. Then, at first the phase was changed using a voltage step size of 0.3 V (cf. Fig. 2), and the step size was lowered to 0.1 V until a minimum was reached. Next, the attenuator was iteratively adjusted using an initial step size of 0.05 V (cf. Fig. 3). In subsequent iterations, a step size of 5 mV was applied until the acquired signal intensity was below the specified threshold. In this preliminary implementation, the total time for one feedback cycle was 50 ms. In the fast CEA implementation (cf. Methods, CEA MRI Measurements), the system was updated every 10 ms based on the input from a hard pulse segment integrated in the RF pulse (Supporting Fig. S1).

To demonstrate the effectiveness of the analog cancellation system with real-time dynamic feedback, the coils were loaded with a human hand positioned horizontally above the Rx coil. The volunteer rotated his hand three times during the CEA calibration sequence, which changed the loading of the CEA coil system, resulting in an increased Tx-induced leakage signal.

To demonstrate the temporal stability of the system during MR imaging, the coupling of the unloaded coil system was first minimized with the calibration sequence, and then a concurrent excitation and acquisition sequence with 100 radial spokes was acquired with TR = 6 s using a smooth-chirp excitation with 16 kHz sweep range. Measurements were repeated with zero RF voltage to analyze the noise stability – thereafter, the standard deviation of the acquired signal was calculated for each repetition. As the reference receive noise floor, an acquisition was performed when the Tx cables were unplugged.

### CEA MRI Measurements

To test the system, an imaging experiment was performed with a phantom consisting of 8 cylindrical tubes, each filled with a solution of 0.5 g/l NiSO_4_. Additionally, two smaller tubes filled with 1 g/l and 2 g/l CuSO_4_ solutions were attached using a plasticine based industrial dough. A 3D radial CEA imaging sequence with continuous gradient ramp^30^ was applied with the following parameters: 60,000 radial spokes, 512 data points per spoke, TR = 8.2 ms, total scan time = 492 s, acquisition window = 4.1 ms, maximum gradient strength = 4 mT m^−1^, chirp RF pulse with 16 kHz sweep. The chirp pulse amplitude was smoothed by a sinusoidal function at the first and last 10% of the complete pulse duration^41^ to avoid distortions in the acquired signal due to sudden onset of the RF signal. The peak power at the output of the power amplifier was measured as 17.3 mW. The effective flip angle was estimated as 0.48°^24^.

The performance of CEA for imaging of ultra-short T2* samples was compared to a conventional UTE sequence using iron-oxide contrast agent solutions. Six plastic tubes filled with contrast agent RESOVIST (Bayer Schering Pharma AG, Leverkusen, Germany) corresponding to Fe concentrations of 0.02, 0.029, 0.038, 0.056, 0.071, and 0.1 M were imaged. For the UTE acquisition, the following parameters were used: TE = 70 µs; TR = 2.6 ms; flip angle = 2°; averages = 10; FOV = 320 mm; BW = 1488 Hz/px; radial spokes = 64000, number of points per spoke = 160; total scan time = 1664 s. With CEA, the following parameters were used: RF bandwidth = 128 kHz; TR = 2.6 ms; effective flip angle = 0.12°; averages = 10; FOV = 320 mm; acquisition time = 2.1 ms; radial spokes = 64000, number of points per spoke = 320; total scan time = 1664 s. Signal-to-noise ratio (SNR) values were calculated and scaled to account for the differences in imaging parameters^42^. A UTE pulse sequence with the same parameters was also used to measure T_2_* of the phantoms for TE = {70, 100, 120, 140, 160, 180, 200, 500} µs. T_2_* of the phantom with 0.1 M was estimated by assuming a linear relationship between the Fe concentration and 1/T_2_*.

To demonstrate *in vivo* applicability, CEA data of the wrist, head and ankle were acquired. The wrist of a healthy volunteer was imaged using the same parameters as in the NiSO_4_/CuSO_4_ phantom image. As the initial values for DC voltages of the attenuator and the phase shifter, values from the phantom setting were used. Both phantom and *in vivo* CEA images were compared to a high resolution gradient echo (GRE) image as an anatomical reference (TR/TE = 10/2.85 ms, flip angle = 15°, FOV = 200 mm, acquisition matrix = 512 × 512, 32 slices with 1.88 mm thickness, total scan time = 82 s). The peak power at the output of the power amplifier was measured as 26 W. For head imaging, a CEA protocol with 2.4 ms TR and 2 ms-long hyperbolic-secant (HS8) RF pulse sweeping through 64 kHz was used, and data were compared to a 3D radial UTE sequence. The UTE parameters were TR/TE = 3.6/0.07 ms; FOV = 320 mm; flip angle = 4°; RF pulse duration = 0.06 ms; 160 samples per spoke and 80,000 spokes; total scan time = 288 s. In CEA head MRI, 80,000 spokes were acquired with 5 averages, corresponding to a total scan time of 960 s, and the effective flip angle was estimated as 0.27°. Finally, a fast CEA protocol with 250 mm FOV and 1.57 mm nominal resolution was prepared using 1 ms-long HS8 RF pulse with 128 kHz sweep range for CEA MRI of the right ankle of a healthy volunteer. Sweep range was chosen as 128 kHz. TR was set to 1.1 ms accounting for the 50 µs intentional delay between the start of the ADC and the RF pulse, as well as the ramp-up time of the gradients. Total scan time of 80,000 spokes was 1 min 28 s.

An additional performance test was conducted to measure the acoustic noise performance of the CEA pulse sequence. A digital sound level meter (DSL 331, Tecpel Co. Ltd.,Taiwan) was placed at a distance of 2 m to the magnet, with the calibrated microphone pointing towards the magnet bore. The imaging sequences were compared to the background noise level in terms of acoustic pressure levels.

### Data processing

Even with an optimal analog cancellation, some additive residual transmit leakage in the received signal is observed that has a frequency-dependent amplitude modulation. Decoupling is generally maximal at the frequency at which the analog cancellation system was calibrated, and it is less efficient at off-resonance frequencies. The frequency-dependent amplitude behavior of the residual leakage was approximated by a linear fit at both sides of the center frequency of the modulation in chirp pulse. The Tx signal acquired with the PUC was also phase-corrected by fitting a quadratic polynomial to the phase profile of the received signal. For HS8 pulses, the leakage signal was modeled as the theoretical pulse function multiplied by superposition of modulations that are quadratic and linear functions of time, and a phase constant. An optimization algorithm based on simulated annealing was run to minimize the total signal power. The parameters were than manually adjusted to extract the MR signal by comparing the data set to a calculated MR signal (i.e. numerically solving the Bloch equations) and the residual leakage pair. Then, the residual leakage was subtracted from the received signal to obtain the corrected signal, ${s}_{{MR}}(t)$.

For image reconstruction, ${s}_{{MR}}(t)$ is assumed to be a convolution of the FID response from the excited spins $h(t)$ with the RF pulse ${u}_{{Tx}}(t)$^22^ (cf. also Fig. 6 in ref.^28^), which is a valid assumption for small flip angle excitations^43^, which is written in discrete notation as:

2 $${s}_{MR}[n]=h[0]{u}_{Tx}[n]+h[1]{u}_{Tx}[n-1]+h[2]{u}_{Tx}[n-2]+\cdot \cdot \cdot .$$

To reconstruct $h$, the following equation in matrix form needs to be solved:

3 $${{\bf{s}}}_{{\bf{MR}}}=\mathrm{UH}{\boldsymbol{,}}$$

which leads to the Penrose pseudo-inverse formulation:

4 $${\bf{h}}={({{\bf{U}}}^{\ast }{\bf{U}})}^{{\boldsymbol{-}}1}{{\bf{U}}}^{\ast }{{\bf{s}}}_{{\bf{MR}}}$$

The length of the impulse response, $h$, is determined experimentally by checking the root-mean-square error. This calculation is repeated for each acquired radial spoke. A k-space center shift correction was applied based on the cross correlation of the opposite radial spokes to account for sub-pixel shifts in the calculated FID similar to the gradient delay calculation used in conventional radial imaging^44^. Finally, a 3D set of non-uniformly sampled FID data was mapped on a Cartesian grid using Kaiser-Bessel interpolation^45^ and Fourier transformed to obtain the image data. All calculations were implemented in MATLAB 9.0 (The MathWorks, Inc., Natick, Massachusetts, United States).

### Data availability

The datasets measured and analyzed during the current study are available from the corresponding author on reasonable request.

### Ethics

*In-vivo* imaging experiments were carried out in accordance with the protocol for volunteer MR experiments which was approved by the institutional review board (Ethikkommission) of Albert-Ludwigs-University of Freiburg. Written informed consent was obtained from the subject prior to the MR examination.

## Results

### Analog cancellation performance tests

**Figure 4:**
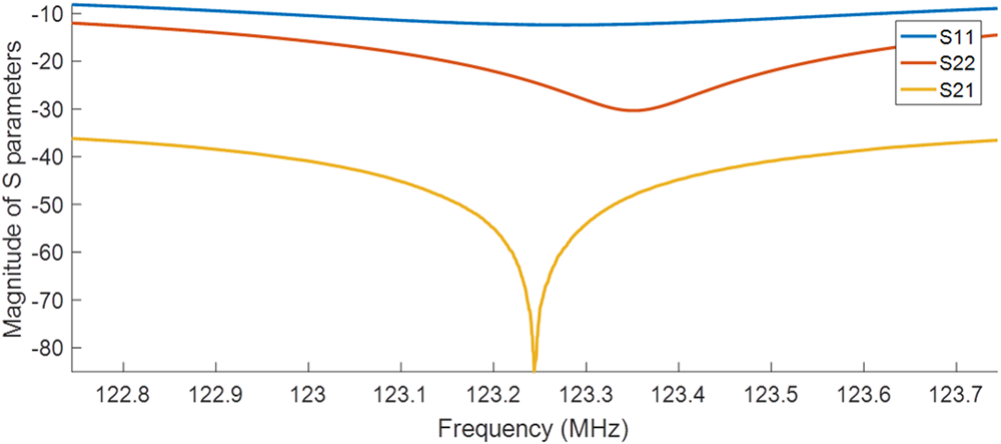
Network analyzer measurement results for the manually tuned analog cancellation circuit using the experimental coil setup.

In Fig. 4, measurements with network analyzer (ZVB4, ROHDE&SCHWARZ, Munich, Germany) show that the combination of the analog cancellation system and geometrical decoupling yields 60 to 85 dB decoupling over a 48 kHz band around the center frequency, of which 48.6 ± 12 dB is provided by the analog cancellation system alone. Input and output reflection coefficients, |S11| and |S22|, are larger than 12.5 dB over a frequency range of 200 kHz, which is larger than the acquisition bandwidth used later for imaging by about 16 kHz.

**Figure 5:**
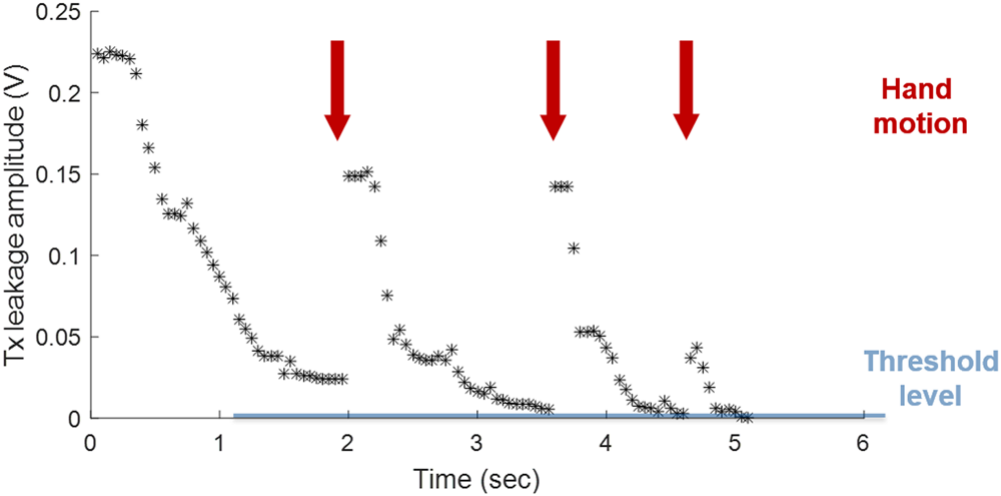
Voltage level of the residual Tx-induced leakage signal during automated feedback cancellation. Arrows show the instants when the hand position was changed and re-iteration was triggered.

In the 3 T MRI system, dynamic decoupling performance was tested under real time feedback operation. In the experiment with the moving hand, transmit leakage increased by about 0.05–0.15 V after each hand motion (Fig. 5). With the real-time feedback system, initial decoupling took 40 iterations or 2 s to converge, and in other experiments at maximum 100 iterations were needed. Subsequently, fewer iterations were required to re-adjust the parameters after the position of the hand was changed intentionally (Fig. 5, arrows). The performance was not affected when the threshold was lowered from 78 dB to 89 dB on-resonant isolation measured over a 2 kHz bandwidth.

The stability and noise performance of the analog cancellation system are shown in Supporting Fig. S2. The temporal signal variation in the unloaded coil amounted to 0.2% of the mean of the signal. In the noise measurement the receive noise increased by up to 9.7% from the noise floor when the transmit coil was operated with 0 V input.

### CEA MRI measurements

**Figure 6:**
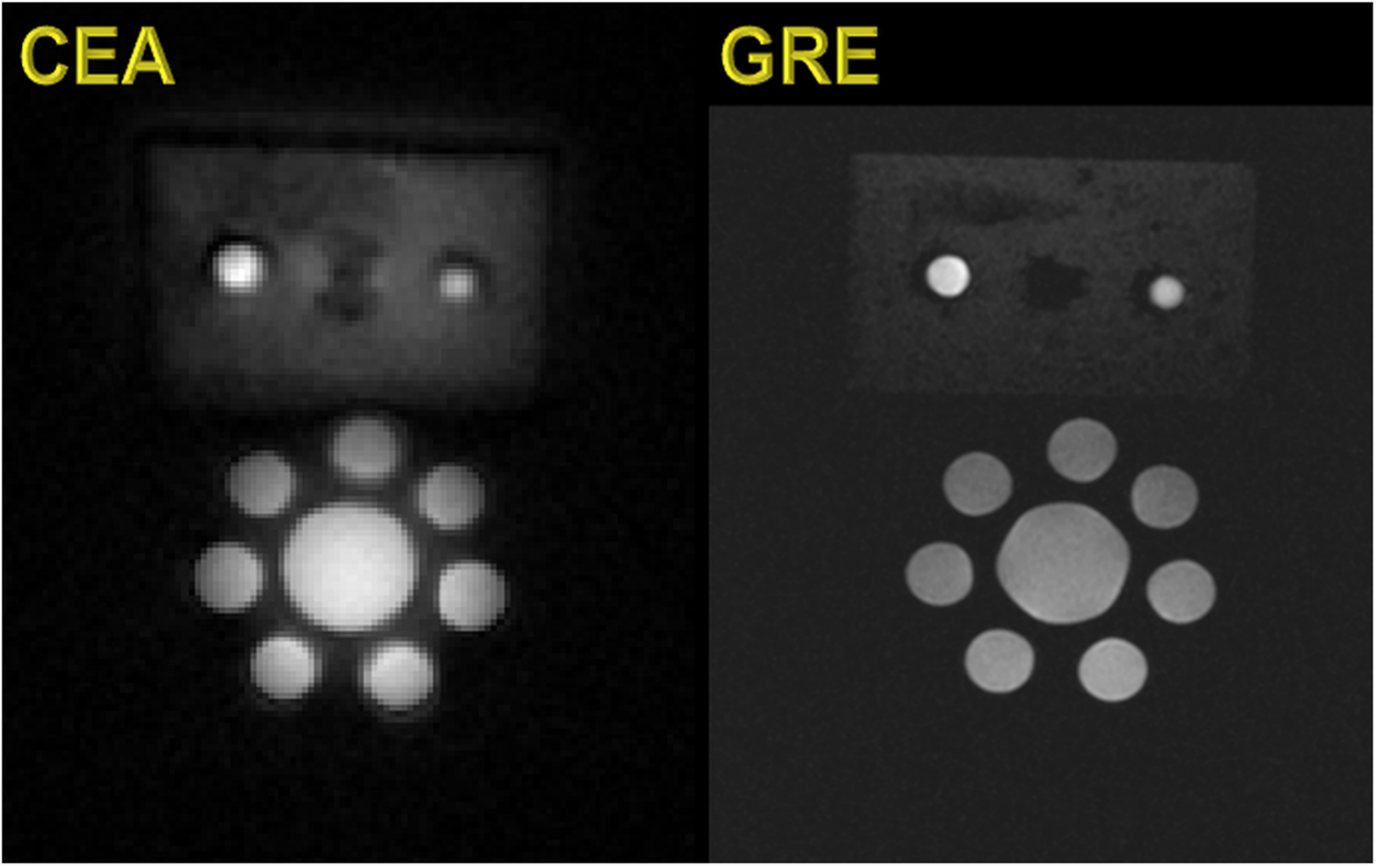
Coronal slice from the 3D CEA MR image of the tube and rubber phantoms (left), and GRE image as the structural reference (right).

**Figure 7:**
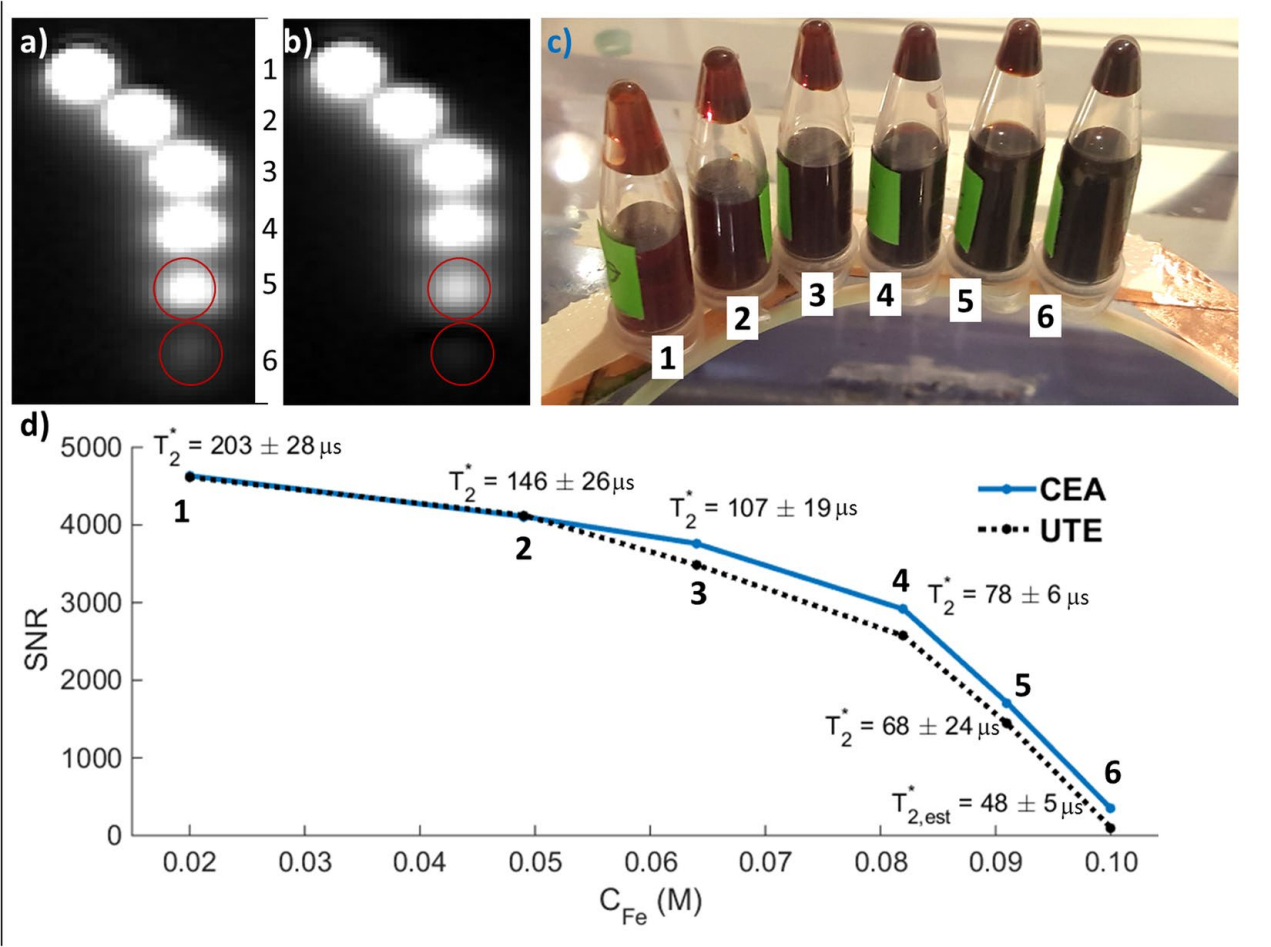
Comparison of CEA and UTE in MRI of iron-oxide solutions. A coronal slice from the images acquired with (**a**) CEA and b) UTE. (**c**) A photo of the iron-oxide phantoms with different concentrations placed on the CEA coil form, which is made of glass. (**d**) SNR comparison for various Fe concentrations and T_2_* values measured using the UTE sequence. The last T_2_* value, i.e., for phantom 6, was estimated by assuming linear dependence of 1/T_2_* on C_Fe_.

In Fig. 6a a comparison is shown of a coronal slice of the phantom acquired with CEA and GRE. The phantom tubes are clearly visible in both acquisitions (bottom), but the surrounding rubber (top) has 3.5 fold higher signal-to-noise ratio (SNR) with CEA than with GRE. The comparison of the signals from the iron-oxide samples show that UTE can hardly detect the signal in the vial with a T_2_* shorter than 50 µs, whereas CEA is still capable of visualizing this vial with only a 4–5fold reduced signal intensity (Fig. 7).

**Figure 8:**
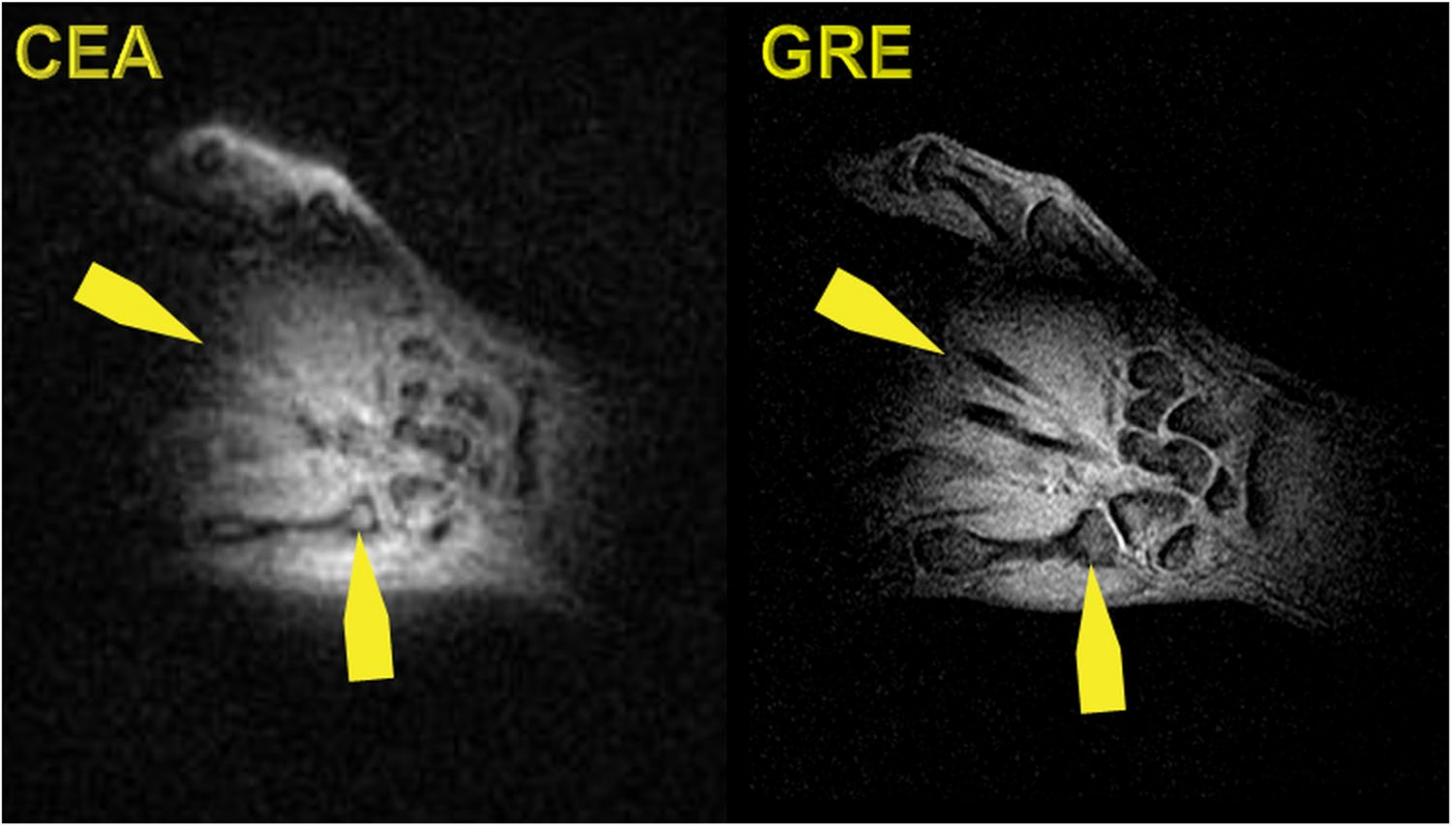
Coronal slice from the 3D CEA MR image of the healthy volunteer wrist (left), and GRE image as the anatomical reference (right).

Figure 8 shows a coronal slice of the 3D data of the human wrist acquired with CEA and GRE. Slices are not identical since the experiments are conducted at different times. The wrist bones are seen with a higher intensity in the CEA image, and extensor finger tendons, which are not represented in the GRE image, provide a higher signal intensity in CEA than in GRE. Carpal bones also have a higher signal in a CEA image even in trabecular bone. The overall contrast to noise is higher in GRE due to intrinsic T_2_* weighting.

**Figure 9:**
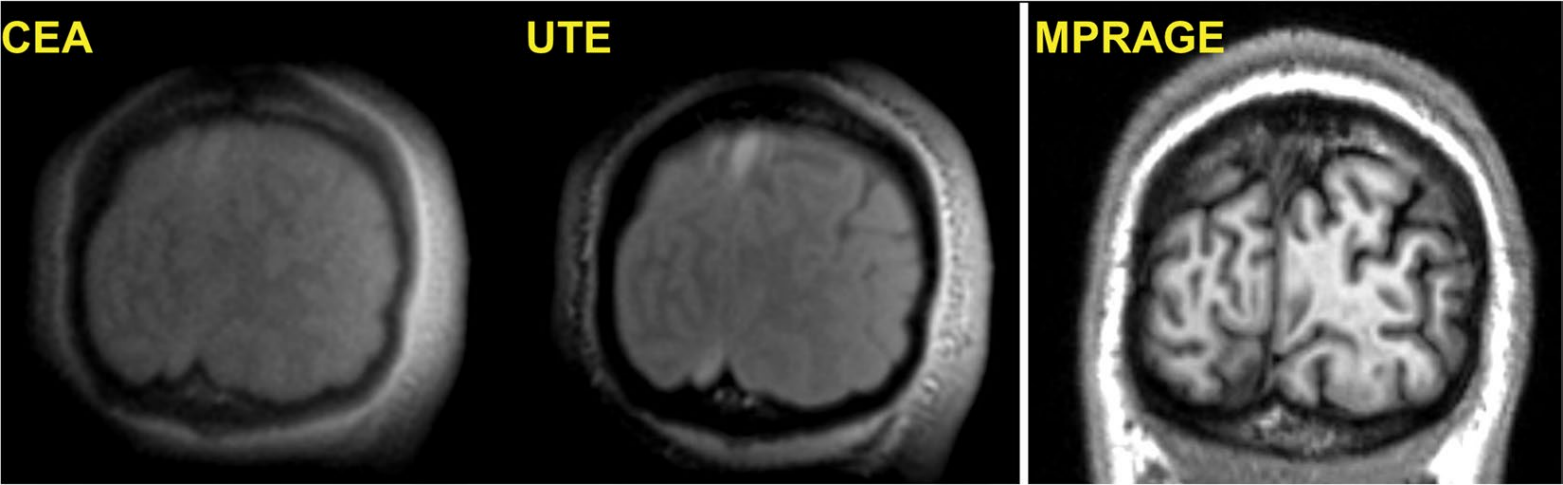
Coronal slices from the 3D CEA and UTE image data of a human head registered on a previously acquired T_1_-weighted MPRAGE data set as the anatomical reference.

In Fig. 9, coronal slices of the CEA and UTE images of a human head are shown. The data is co-registered to a previously acquired T_1_-weighted MPRAGE data set of the same volunteer. Compared to UTE, CEA has lower SNR and minor artifacts at the edges. The contrast-to-noise ratios for cortical bone/white matter and cortical bone/gray matter for manually selected region of interests (ROIs) are similar (23.9 and 25.9 for UTE, 22.9 and 25.0 for CEA, respectively). The SNRs for UTE/CEA images are calculated as 1058/755, 810/559, and 669/438 for cortical bone, white matter, and gray matter tissue, respectively at the manually selected ROIs. A lower flip angle of CEA reduced the in-flow effect, which resulted in decreased signal intensity in the sagittal sinus.

**Figure 10:**
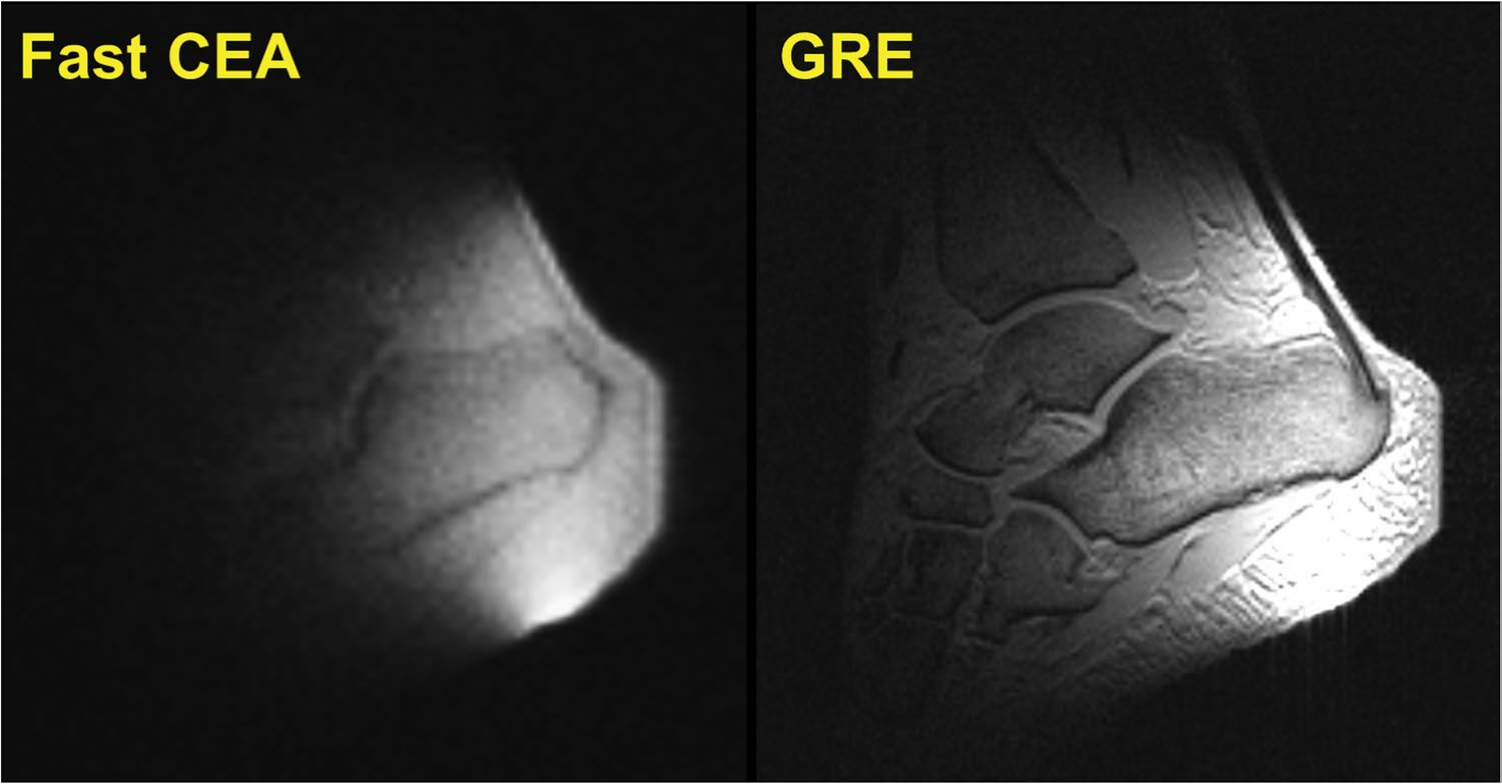
A sagittal slice of the fast CEA image of a human ankle (left), and the GRE image as anatomical reference (right).

A sagittal slice from the fast CEA image of a human-ankle is shown in Fig. 10. The main anatomical structures agree with the GRE image. The Achilles tendon is clearly visible in the CEA image, whereas it is represented as signal void in the GRE image.

Acoustic noise measurements showed that for a gradient amplitude of 29 mT m^−1^ and a slew rate of 120 mT m^−1^ s^−1^, the acoustic noise of the CEA sequence is 54.8 ± 0.3 dBA which is only 11.2% higher than the background noise level of 49.4 ± 0.1 dBA, whereas the noise for the GRE sequence reached 81.8 ± 0.4 dBA, exceeding the background noise level by 68%. A UTE sequence with the same number of spokes was performed at 78.6 ± 2.0 dBA and at 74.1 ± 1.8 dBA, for maximum gradient strengths of 20 mT m^−1^ and 10 mT/m, respectively.

## Discussion

In this work, the performance of an automated active analog cancellation system was analyzed, and this system has been used to successfully demonstrate CEA MRI on a clinical MR system to obtain phantom and *in vivo* images. The acoustic noise performance, acquisition efficiency, and low power requirements, suggest that CEA is potentially valuable for future MRI applications.

### Automated feedback decoupling system

The analog cancellation system proposed in this work provides a suppression of the leakage signal of about 60 ± 2 dB, which is consistent with cancellation values found in full-duplex radio communication^35^. The combination of geometric decoupling^19^ and the analog cancellation system always achieved a suppression of 89 ± 2 dB, which was also used as a stopping criterion for the automatic feedback system. In clinical MRI systems this is not sufficient, and thus additional digital subtraction during post-processing was necessary to achieve the required total isolation of 100 dB^29^.

Both the analog cancellation circuit, as well as the geometrically decoupled RF coils, show a frequency-dependent response (cf. Fig. 4), so that the amount of decoupling is dependent on the frequency offset. The frequency dependence of the isolation could be decreased using multistage broadband matching circuits^46^. It is possible to apply bandpass filters with specified frequency responses to achieve a flat leakage profile. An effective full duplex system using such filters was presented in Zhou *et al*.^47^. This method can also be adapted to CEA MRI.

Automated feedback was used with up to 20% duty cycle, which can be increased to achieve more iterations. The number of iterations in the gradient descent optimization was less than 100 for all the trial experiments, because suitable initial points were assigned. This approach is simple and does not require a long look-up table that stores many possible phase and attenuation pairs, as reported in Bharadia *et al*.^36^. When the automated decoupling is triggered, the iterations are done within the running sequence, which may result in some of the spokes being useless during decoupling iterations and hence getting discarded from the reconstruction data space. A next step for a more practical implementation is to control the pulse sequence environment for acquisition of the missing spokes during decoupling iterations.

#### Noise

In CEA MRI, Tx noise is one of the most important factors as it directly affects SNR. A maximum deviation of less than 10% from the receive noise floor was measured. Although this measurement suggests that the analog cancellation circuit does not contribute to the system noise significantly, it requires further analysis to relate the system noise to the SNR. Our first proof-of-principle circuit was designed without taking special care to use low noise components, such as varactors and PIN diodes with exclusive noise characteristics. There is therefore still room for improvement of our analog circuit implementation.

Another detrimental factor in CEA is cable-cable coupling, which can result in significant reduction of the decoupling performance, especially if unbalanced shield currents are present. If more than one Rx channel is used (e.g., in PUCs or Rx arrays), the receive circuitry and cables should be well separated both electrically and physically. In our setup, a separate receiver plug was used for the PUC. Additionally, coaxial cables of the whole setup should be shielded externally, or triaxial cables should be used instead.

Inhomogeneous field distributions of the surface coils affect the image quality. On the other hand, volume coils such as the birdcage coil offer better homogeneity. Linearly or circularly polarized volume coils are compatible with the existing setup - the only part that needs to be changed is the geometrical decoupling. With a linearly polarized birdcage transmit coil, a surface coil can be placed at the zero electric field plane, which could provide isolation up to 20 dB^27,48^. In case of a birdcage coil driven in quadrature mode, hybrid couplers serve as isolators. Proper circulators can also provide reasonable passive decoupling.

Full digital cancellation approaches can also be up-scaled to perform in clinical MRI systems as an alternative to the analog cancellation approaches^49,50^. A receiver with high dynamic range is required to avoid saturation and to minimize quantization errors. Comparison of different CEA decoupling strategies will be addressed in a separate study.

### Reconstruction

Chirp RF pulses with sinusoidal modulated edges and hyperbolic-secant pulses were used in our experiments. Due to the frequency-dependent response of the decoupling system, the pulse amplitude was modeled during post processing to remove the residual transmit leakage from the acquired signal. To improve the efficiency of reconstruction, an iterative regularization might be used to calculate the residual leakage by taking the frequency-dependent modulations into account.

Reconstruction in its current state must be improved to perform faster, and to provide artifact-free images. Analytical solutions to the Bloch equations can be utilized to improve leakage-free CEA signal estimation and to describe encoding steps in algebraic form^51^. The algebraic reconstruction approach is preferred when there are missing points in the acquired data as in SWIFT or ZTE^52^. In CEA MRI, the encoding matrix can be extended by columns describing the Tx-induced leakage elements, and the components of the polynomial that describes the frequency dependence can be extracted from the matrix solution.

### Contrast mechanisms

The phantom and *in vivo* images suggest that there is a dominant proton density weighting along with a T_1_ relaxation based contrast which can be deducted from the small CuSO_4_ tube phantoms with different concentrations. The images were compared to a high resolution GRE sequence and a 3D UTE sequence. The proof-of-principle CEA images are promising for representing tendons with high signal intensity, compared to GRE images where the tendons are devoid of signal. As expected, CEA could visualize samples with shorter T2* than UTE, and even if shorter echo times could be used in UTE, the fundamental limit of the dead time between RF excitation and acquisition would still remain. The contrast behavior of the UTE and CEA, on the other hand, are very similar. Effective flip angle of CEA can be increased by applying a stronger RF pulse if the transmit noise is eliminated. To manipulate the contrast in CEA, preparation pulses can be applied. CEA sequence is preceded by a preparation module (e.g., 90°-TE-(−90°) for T_2_-weighted CEA, or 90°/180° for T_1_-weighted saturation or inversion recovery CEA), or with higher CEA flip angles even steady state contrasts can be established. This, however, requires a modification of the CEA system to enable switching between low power sweep pulses and high power saturation/inversion pulses. To perform these high power excitations in the preparation, the existing whole body transmit coil can be used, and the CEA coils would be detuned. Dependence of the signal contrast on the frequency sweep parameters has to be studied explicitly.

### Receiver system

With increasing decoupling, the probability of introducing quantization errors in the received signal is reduced. Therefore, the task of the decoupling is not only to reduce the residual leakage down the Rx dynamic range, but also to make an efficient use of ADC dynamic range to increase image quality. Considering the frequency-dependent nature of the residual leakage, ADC bits that define the ‘clean’ signal are always limited, and as a result also the SNR. In order to overcome this problem, real-time cancellation systems are required that can provide higher isolation and flat residual leakage profile, and which can respond to the loading changes at every TR. This way, it is possible to benefit from full ADC dynamic range for more efficient encoding of the MR signal.

In future refinements of this setup, the possible use of this system with receive arrays could be considered. For a receive array configuration, one possible setting is to use a separate analog cancellation circuit for each channel. However, in this configuration, couplings among the coils and the cables will complicate the solution, and assigning a single phase and amplitude scale for each channel will not be as simple as for the single coil case. A more advanced solution would be to design version of the current automated feedback decoupling that is extended to the number of Rx elements. An optimization algorithm could then search for the required attenuation and phase settings.

Elimination of auditory noise increases patient comfort, so that longer acquisitions might become feasible, communication with the patient is facilitated, and pediatric patients become more relaxed. Passive noise protection with ear plugs can reduce the unpleasantness of an MRI exam, but as McJury *et al*. pointed out, such noise control techniques fail at suppressing low frequency components, where peak MR imaging sounds are generated, and passive protection do not eliminate acoustic noise conducted through bone^53^. A by-product of the silence is the suppression of eddy currents, as these are caused by rapid gradient switching. Eddy currents can shift the actual k space locations and cause unwanted phase accumulation, leading to a wide range of image artifacts in MRI and distorted line shapes in spectroscopy applications. Compensation of such effects by hardware or post processing is a challenging task^54^.

## Conclusion

CEA MRI with dynamic analog cancellation is an initial step to demonstrate *in vivo* human imaging. CEA is time efficient due to the automated feedback decoupling operation; furthermore a reduction in decoupling due to motion or changes in loading, can be compensated in a short time. With CEA, the signal of ultrashort-T_2_* species become detectable, which will open new imaging possibilities, such as assessing lung parenchyma at 3 T and higher, or measurements of protein content without magnetization transfer, which are, however, beyond the capabilities of the current prototype setup. Considering the advantages of nearly 100% acquisition efficiency, elimination of the gradient switching noise, and extremely low peak RF powers, CEA MRI is potentially a promising tool, for various clinical applications including musculoskeletal imaging, dental imaging, connective tissue and myelinated neurons.

## Electronic supplementary material

Supplementary Information
