# Supplementary material for: In vivo MRI with Concurrent Excitation and Acquisition using Automated Active Analog Cancellation
Source: Sci Rep. 2018 Jul 13;8:10631. doi: 10.1038/s41598-018-28894-w (PMC6045667; doi:10.1038/s41598-018-28894-w)
Supplement: Supplementary file 1 — Supplementary Information [file 41598_2018_28894_MOESM1_ESM.pdf]

In vivo MRI with Concurrent Excitation and Acquisition using Automated Active Analog Cancellation

Ali Caglar Özen[1, 2,*], Ergin Atalar[3], Jan G. Korvink[4] , Michael Bock[1]

[1]Dept. of Radiology, Medical Physics, University Medical Center Freiburg, Freiburg, Germany

[2]German Cancer Consortium Partner Site Freiburg, German Cancer Research Center (DKFZ), Heidelberg, Germany

[3]Dept. of Electrical and Electronics Engineering, Bilkent University, Ankara, Turkey

[4]Inst. of Microstructure Technology, Karlsruhe Institute of Technology, Karlsruhe, Germany

*Correspondence to Ali Caglar Özen (ali.oezen@uniklinik-freiburg.de)

Fast CEA implementation

Another method for measuring Tx-induced leakage for dynamic cancellation uses short (10 µs) hard pulse acquisition at the end of each TR embedded in the hyperbolic-secant pulse (HS8) arrays (Supporting Fig. S1). During the fast CEA implementation, this method was used. The data points for the calibration pulses are separated from the actual imaging data and used to determine amplitude and phase parameters iteratively for the analog cancellation unit.

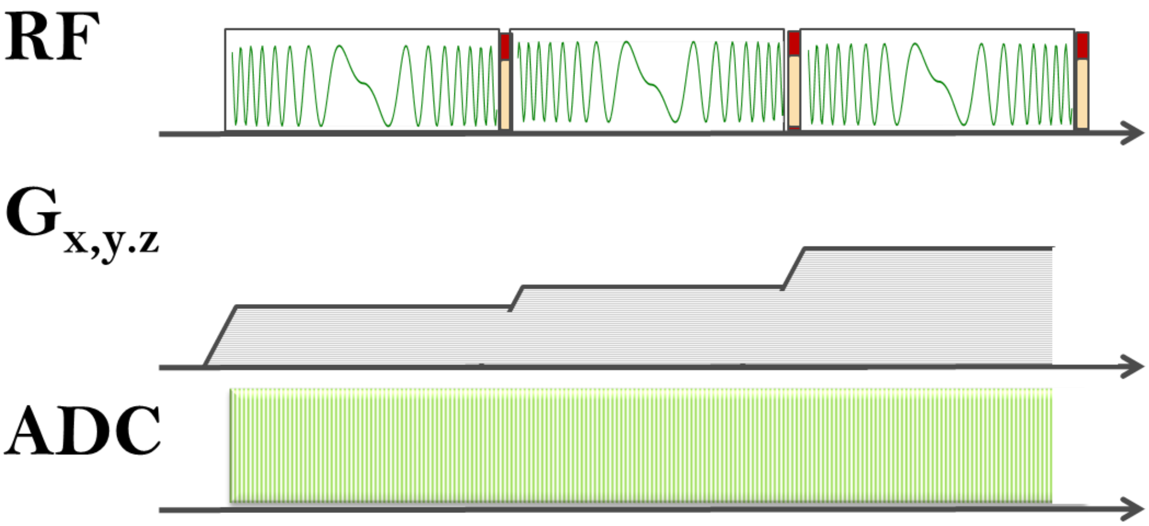

Supporting Figure S1: CEA pulse sequence with embedded calibration pulses (red/yellow). The data points for the calibration pulses are separated from the actual imaging data and used to determine amplitude and phase parameters iteratively for the analog cancellation unit.

Stability and noise performance of the analog cancellation system

The stability and noise performance of the analog cancellation system are shown in Supporting Figure S2. The temporal signal variation in the unloaded coil amounted to 0.2 % of the mean of the signal. In the noise measurement the receive noise increased by up to 9.7 % from the noise floor when the transmit coil was operated with 0 V input.

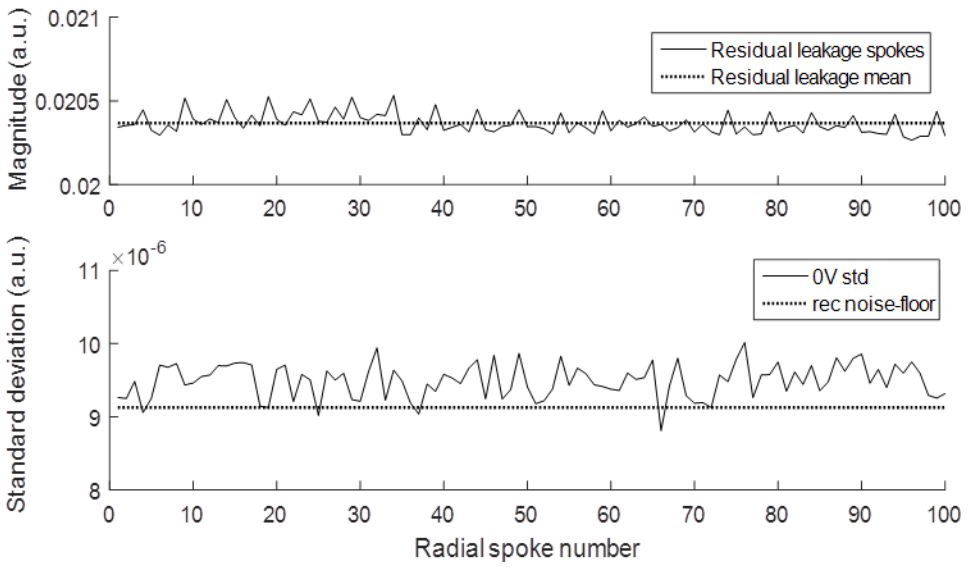

Supporting Figure S2: Stability and noise checks of the analog cancellation system during MRI. Upper part shows the difference between the residual leakage of two spokes and the mean of all the spokes for maximum and minimum cases. Maximum of 0.2 % magnitude deviation from the mean was observed. At the middle of the sweep, MR signal dependent modulations starts to be seen. Bottom part shows the noise standard deviations for each spoke compared to the mean of all spokes and the receive noise floor. Maximum of 9.7 % increase from the receive noise floor was observed.
